# Implant Placement Following Crestal Sinus Lift with Sequential Drills and Osteotomes: Five Years after Final Loading Results from a Retrospective Study

**DOI:** 10.3390/jfb12010010

**Published:** 2021-02-04

**Authors:** Aurea Immacolata Lumbau, Silvio Mario Meloni, Marco Tallarico, Luca Melis, Giovanni Spano, Edoardo Baldoni, Alba Koshovari, Milena Pisano

**Affiliations:** 1 School of Dentistry, University of Sassari, 07100 Sassari, Italy; alumbau@uniss.it (A.I.L.); melonisilviomario@yahoo.it (S.M.M.); giovanni.spano@aousassari.it (G.S.); baldoni@uniss.it (E.B.); 2 Private Practice, 07100 Sardinia, Italy; lucamelis.od@gmail.com (L.M.); milenapisano@yahoo.it (M.P.); 3 Department of Implantology and Prosthetic Aspects, Aldent University, 1022 Tirana, Albania; alba.koshovari@ual.edu.al

**Keywords:** dental implants, sinus lift, crestal approach, osteotomes, atrophic maxilla

## Abstract

The aim of this retrospective study was to clinically evaluate the five-year outcomes of implants placed following a combined approach to the sinus, consisting of sequential drills and osteotomes. Medical records of patients with implants placed in combination with crestal sinus lift using sequential drills and osteotomes, with a residual alveolar bone crest between 4 to 8 mm, and a follow-up of at least five years after final loading, were evaluated. Outcomes were implant and prosthetic survival and success rates, any complication, and marginal bone loss. Data from 96 patients (53 women and 43 men; mean age 54.7 years; range 23–79 years) were collected. A total of 105 single implants were analyzed. After five years of function, two implants were lost and two prostheses failed. No major biological or prosthetic complications occurred. At the five-year examination, the marginal bone loss was 1.24 ± 0.28 mm. Within the limitations of this retrospective study it can be concluded that implants placed following a combined approach to the sinus consisting of sequential drills and osteotomes seem to be a viable option for the treatment of posterior atrophic edentulous maxilla.

## 1. Introduction

Maxillary sinus lift with one or two staged implant installation is a suitable technique for both complete and partial edentulous patients [1]. In these patients an alveolar ridge atrophy, both vertical and horizontal, may occur due to the maxillary sinus pneumatization, requiring different surgical approaches prior, or in combination with implant placement [1,2,3,4,5,6,7,8,9,10,11,12]. Classical sinus augmentation procedure was first described by Tatum Jr. in 1974 [13], and a few years later by Boyne and co-authors [14]. According to this approach, named a lateral sinus lift, a lateral bone window is opened, and bone substitute is grafted under the elevated Schneider membrane. The most-reported graft material is xenograft mixed or not with autogenous bone. Use of stem cells has been also proposed, but according to a recent study, it seems not to significantly improve the implant survival rate, and/or the efficacy of bone regeneration following sinus lift procedure [15,16].

In addition, the Schneider membrane can be approached through the alveolar bone crest [17]. This latter approach, named crestal approach to the sinus (or transcrestal approach), was first described by Summers in 1994 [18], and it is completely performed by osteotomes that allow the cortical bone to fracture and the sinus membrane lifting by means of gently grafting material pressure. Several modifications of the crestal approach have been described in literature after Summers’ first report [18]. Cosci et al. modified the summers technique by using atraumatic lifting equipment, with the aim to reduce the risk of sinus membrane perforation, and also to allow a one-stage approach [19]. Furthermore, a less invasive technique for the sinus floor elevation, by using piezoelectric surgery based on a dedicated surgical kit, was described by Wallace and co-authors [20].

Several dental implant designs and materials have been developed in the past decades, aiming to improve the long-term stability of implant-supported restorations. Both commercially pure titanium (cpTi) and Ti-6Al-4V are highly satisfactory materials, and they give clinical success rates of up to 99% at 10 years [21]. Better and co-authors [22], and later, Tallarico and co-authors [23,24], described a minimally invasive staged approach performed by using a dedicated dental implant that allows the elevation of the Schneider membrane through hydraulic pressure, and the simultaneous positioning of a flowable bone substitute. Using this technique, sterile saline is injected through the hollow body of the implant to safely lift the sinus membrane. The method is a modified hydraulic approach that comes from the sinus condensation technique described by Chen and Cha in 2005 [25]. Xhanari and co-authors [26] recently compared in a randomized controlled trial crestal versus lateral approaches. The conclusion from this research was that both techniques produced successful outcomes, but the crestal technique required less surgical time and was preferred by patients. Same stable results can be obtained also using hydraulic pressure and conventional implants in a staged approach [27]. However, the main limitation of this randomized controlled trials is the short term follow-up.

The aim of this retrospective study was to clinically evaluate titanium dental implants placed following a combined approach to the sinus consisting of sequential drills and osteotomes, after five years of function.

## 2. Materials and Methods

This research was designed as a retrospective cohort study. The study was approved by the Ethics Committee of the Aldent University, Tirana, Albania (Protocol number 2/2020). A retrospective chart review of previously collected data, including documents, pictures, and radiographs of patients who received at least one transcrestal sinus lift with combined drills and osteotomes approach, treated from April 2009 to January 2014, was carried out. All the surgical procedures were performed by the same oral surgeon in a private clinic. All the restorations were performed by the same dentist and dental technician. This study was conducted according to the principles embodied in the Helsinki Declaration of 2013. At the time of implant placement, all the patients were informed about clinical procedures, materials to be used, benefits, and potential risks and complications, and their written informed consent was obtained for the performed procedures. Patients’ data were anonymized; hence, this research involved existing records that contain non identifiable data about the treated patients.

Medical records of patients aged 18 to 90 years old at the time of treatment, restored with at least one implant in the posterior maxilla, with a residual alveolar bone crest from 4 to 8 mm, evaluated at the computer tomography (CT) or cone beam CT (CBCT) scans, were screened for inclusion. Only implants with readable radiographic images taken at implant insertion one and five years after final prosthesis were investigated. Exclusion criteria were reported in Table 1.

**Figure 1:**
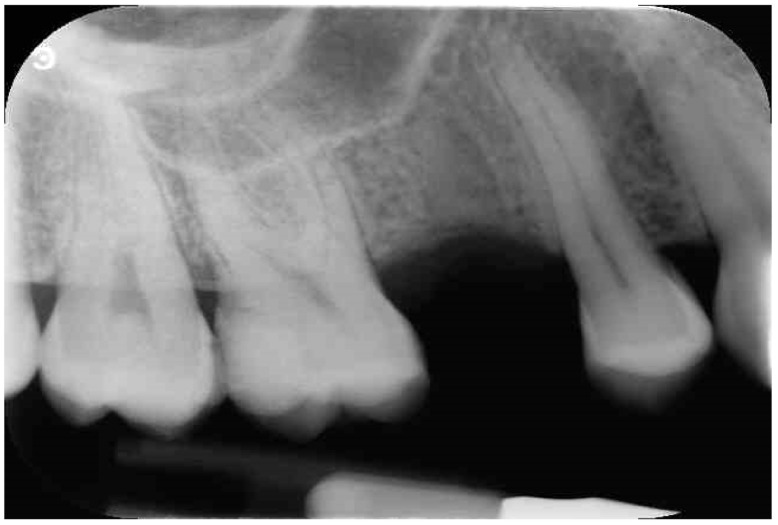
Preoperative periapical X-ray.

**Figure 2:**
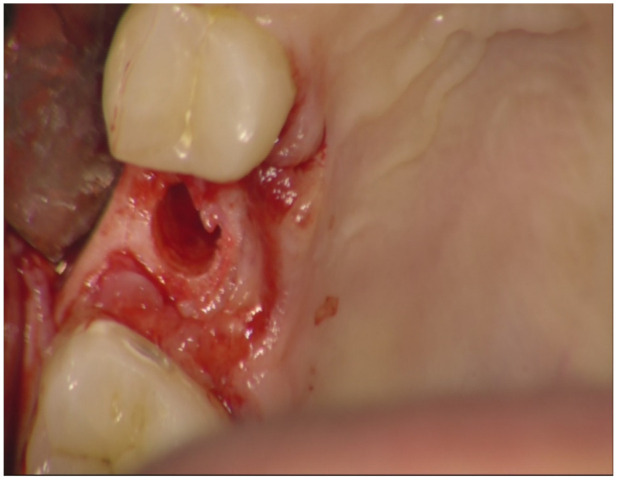
Recipient bed after drill preparation. Occlusal view.

**Figure 3:**
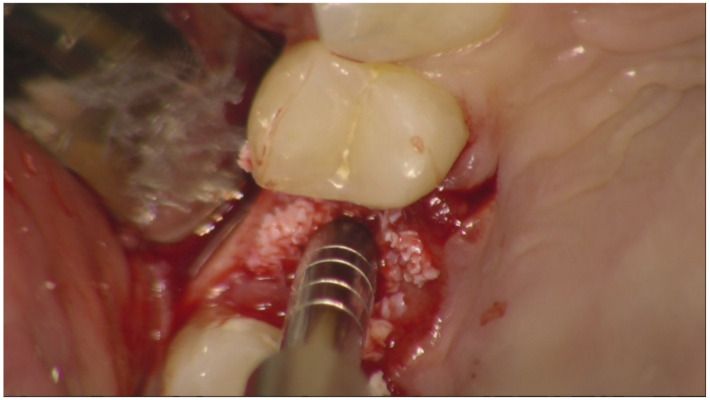
Osteotome crestal preparation. Occlusal view.

**Figure 4:**
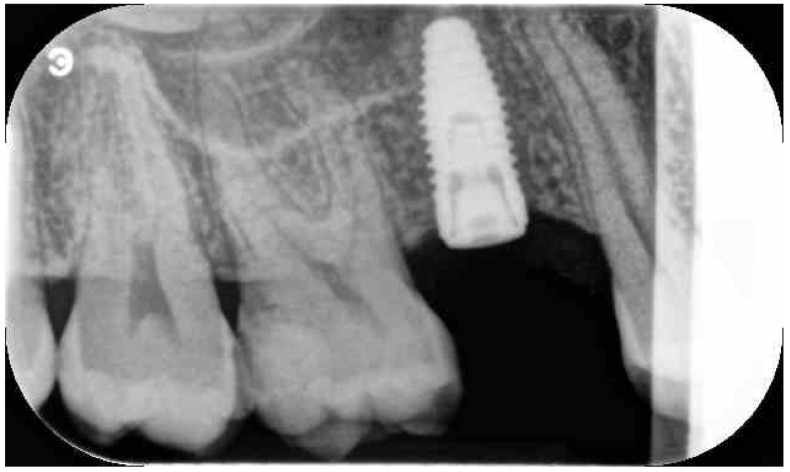
Periapical X-ray immediate after implant installation.

Before surgery, all patients underwent professional oral hygiene with periodontal treatment and supportive therapy, if needed. At the end of the prevention phase, the patients were moved to a careful home and professional oral-hygiene maintenance. Radiographic examination, including periapical radiographs (Figure 1), and computer tomography or CBCT scans, were also performed. All the implants were placed according to a predefined protocol, which includes antibiotic prophylaxis with amoxicillin clavulanate 1 gr every 12 h for six days, starting from the day of the surgery, following by a rinse with 0.2% chlorhexidine (CHX) solution for 1 min. Local anesthesia was performed with a 4% solution of articaine with epinephrine 1:100,000. All the implants were inserted after crestal and intrasulcular incisions performed to raise a mucoperiosteal flap. All the implants were 4.3 mm of diameter (Nobel Replace CC PMC Tapered, Nobel Biocare, Zurich, Switzerland), with 0.75 mm of machined collar. The implant sites were prepared using the lance drill of 1.5 mm of diameter to sign the implant position. Then the twist drill of 2 mm of diameter was used to reach the sinus floor cortex with a drill stop positioned 0.5 mm below the maxillary sinus floor (working length), estimated by using the CT or CBCT scan. Finally, the narrow platform drill (made for 3.5 mm diameter implants, Figure 2) was used at the working length to underprepare the implant recipient site. A collagen matrix (Condress, Smith and Nephew, Agrate Brianza, Italy) was inserted in the prepared site and then the sinus floor was fractured with a calibrated osteotome of 3.5 mm of diameter (Nobel Biocare). At this point, graft material consisting of 0.5 g. of deproteinized anorganic bovine bone, in small microgranules of 0.25–1 mm (Bio-Oss, Geistlich Pharma, Switzerland), mixed with sterile saline, was compacted into the sinus using the same osteotome, up to the working length (Figure 3). At the end of this procedure, all the implants were inserted according to a one-stage protocol [28,29], reaching a primary implant stability from 30 to 50 Ncm. All the implants were 8 to 10 mm of length (Figure 4), depending of the residual bone height. After implant placement, all the patients received oral and written recommendations about the correct maintenance, oral hygiene (i.e., mouthwash 0.2 CHX solutions twice a day, no brushing implant areas), and soft diet. Patients were also instructed to avoid any increase of the intrasinus pressure. The postoperative analgesic treatment was performed with ibuprofen 600 mg, as needed, but a maximum every eight hours for two to three days after the intervention. About two weeks after surgery, sutures were removed.

**Figure 5:**
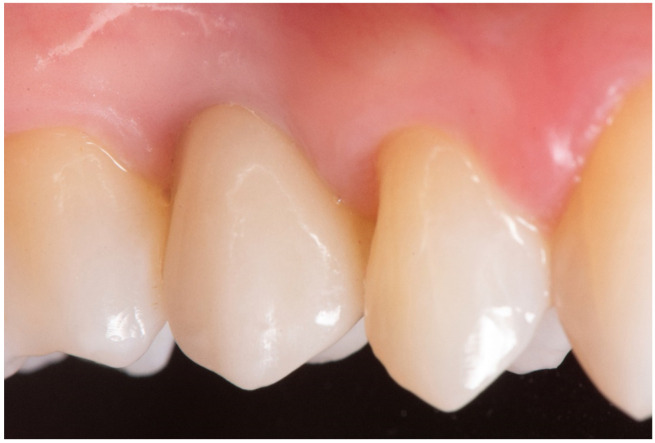
Final crown at 5 years follow-up.

**Figure 6:**
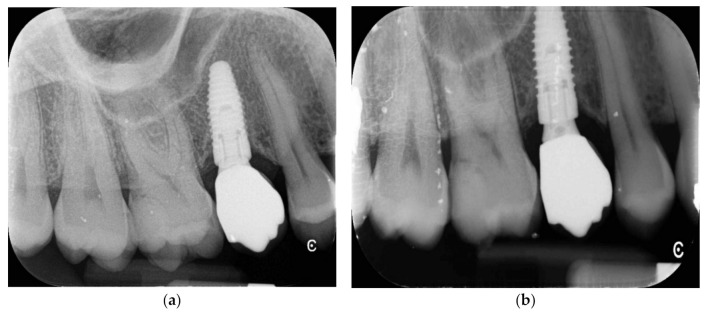
(**a**,**b**) Periapical X-rays at 5 years follow-up.

After six months of undisturbed healing, the second stage surgery was performed and an open-tray impression was taken using a polyether material (ImpregumTM, 3M ESPE, Seefeld, Germany). A temporary screw-retained acrylic crown, (Brent Resin top lign) was delivered one month after the second surgery. Three months later, a definitive impression was made using the same polyether material (ImpregumTM, 3M ESPE) with a customized open tray. Finally, a computer-aided designed/computer aided manufactured (CAD/CAM), screw-retained, zirconia-ceramic (Zirconia multilayer Orodent 1200 MPA) prosthesis was delivered and the occlusion was adjusted. Patients were followed once per year, for five years after loading (Figure 5 and Figure 6a,b).

The primary outcomes were the survival and success rates of implants and crowns. The implant survival and success-rate criteria were a modification of those suggested by Buser et al. [30]. Prosthetic success was assessed following a modification of the evaluation criteria suggested by the California Dental Association (CDA) [31].

The secondary outcomes were any complications and the marginal bone loss. Any mechanical (i.e., fracture of the crown and/or of the restoration material, loosening of the screw) and/or biological (i.e., pain, swelling, suppuration, etc.) complications were reported. Marginal bone levels were measured as the distance between the most coronal margin of the implant neck and the first bone to implant contact. The marginal bone level around the implant was assessed with digital intraoral X-rays, made with periapical long cone paralleling technique (Rinn XCP, Dentsply, Elgin, IL, USA) at the time of implant insertion (baseline), then 12 and 60 months after the final loading. Differences between time points were taken as marginal bone loss (MBL). All readable radiographs were viewed with an image analysis program (DFW2.8 for Windows, Soredex, Tuusula, Finland) on a 24-inch LCD screen (iMac, Apple, Cupertino, CA, USA) and evaluated under standardized conditions (SO 12646: 2004). The software was calibrated for each individual image using the known distance of the implant diameter or its length. Measurements of medial and distal bone crest levels for each implant were obtained with an approximation of 0.01 mm and reported as a mean valor for each patient.

A descriptive analysis was performed by using mean and standard deviation (SD) using SPSS for Mac OS X version 22.0 (SPSS, Chicago, IL, USA). Due to the strictly inclusion and exclusion criteria, data were assumed to be homogeneous, so that the random error was quantified by the standard deviation of the measurements. Moreover, due to the retrospective nature of this research, a priory sample size calculation could not be performed. Nevertheless, a post hoc power calculation was performed for the primary endpoint (implant survival, dichotomous), referring to Pjetursson et al. [32] that reported an implant survival rate of 90% for sites with 4 and 5 mm.

## 3. Results

From April 2009 to January 2014, data from 96 patients (53 women, 43 men; range 23–79 years old; average age 54.7 years) were collected according to the inclusion and exclusion criteria. A total of 105 single-tapered, titanium implants, with anodized surface (Nobel Replace CC PMC, Nobel Biocare), with a diameter of 4.3 mm and 8 or 10 mm length, were initially placed. All the implants were inserted with a surgical motor equipped with external cooling, and set with a maximum implant speed insertion of 40 rpm and 35 Ncm of torque (Bien Air Swiss). Of these, 79 implants were inserted in the first molar position and 26 implants in the second premolar position. Post hoc power calculation of dichotomous primary endpoint (survival rate), for one-sample study was 90.3%.

At the starting position, an average residual crestal bone height of 5.6 ± 0.9 mm was recorded. According to the manufacturer, all the implants were placed at the crest level or slightly above.

Two implants and two temporary prostheses were lost in two different patients, a few weeks after temporary prosthesis delivery, due to lack of osseointegration. Both implants were removed. After four months of healing, both patients underwent the same procedures to replace the failed implants, without any further complications. The cumulative implant and prosthesis survival rate was 98.1% at implant level and 97.9% at patient level. At the five years after final loading follow-up, there were no major biological or mechanical complications. Minor biological complications were experienced in two different patients who developed a peri-implant mucosal inflammation, with positive bleeding on probing, six months after final loading. An improvement in oral hygiene was sufficient to reduce the peri-implant inflammation until complete healing, with no need of any adjunctive surgical or prosthetic procedure. Five years after definitive prostheses delivery, the mean marginal bone loss was 1.24 ± 0.28 mm (Table 2).

## 4. Discussion

This retrospective study aimed to evaluate the five-year after final loading results of implants placed following crestal approach with a combined drills and osteotomes sequence. The main limitations of this study are its retrospective nature and the lack of a control group. Nevertheless, the present study reported a relatively long follow-up, with data of five years after final loading and with a consistent number of examined cases that allowed reaching a post hoc power calculation of 90%.

Both crestal and lateral approaches to graft the sinus aim to obtain a bone reconstruction that allows an appropriate insertion of dental implants [33]. The classical lateral approach to maxillary sinus elevation needs a large mucoperiosteal flap, which influences postoperative morbidity, the higher cost of the procedure, and possible complications (i.e., perforation of the membrane, epistaxis, pain, swelling, bleeding, hematoma, and sinus infections) [34]. Several studies have shown that the crestal technique obtained by osteotomes is less invasive than the lateral window. Moreover, the benefits of the transcrestal approach are the shorter treatment time of the intervention, the shorter healing period, and more predictable, primary stability of the implant [35]. All of this reduces the overall risk of implant failure and/or complications [36]. On the other hand, the complete osteotome approach, as originally described by Summers [19], could present annoying sensations due to the use of a large mallet. With a combined use of drills, osteotomes and tapered implants, as described in the present study, the osteotome is used only once to fracture the cortical bone, and then only gently to compact the graft into the sinus cavity. In this way the annoying use of the mallet is really reduced to only a few seconds. According to Wallace [21], the residual crestal bone height is a discriminating factor between the lateral or crestal approach, affirming that if a residual alveolar bone crest of 3 mm or less is present, a lateral approach must be performed. In this case, the implants will be placed about six months after intervention. On the contrary, if a residual alveolar bone crest of 3 to 6 mm is present a lateral approach with one stage implant installation is recommended. Finally, if there is a residual crest of 7 mm, a transcrestal osteotome approach is suggested. Similar data are also confirmed in a recent randomized controlled trial, opening a larger option for the transcrestal approach [26]. Several studies reported high implant survival rates with a crestal hydraulic approach, even with a residual alveolar bone crest 3 mm high [22,23,24,25]. Nevertheless, this approach is more complex and it needs dedicated surgical tools, and/or dedicated implants, also requiring a medium-to-long learning curve [22,25]. Other systems, recently introduced in the market, seem to reduce the overall complexity, improving the safety of the procedure. Nevertheless, long-term data are still not available [2].

From the brief analysis of the mentioned studies, it seems that despite different approaches to lift the maxillary sinus, the heterogeneity of the techniques and the different indications can cause confusion in terms of indications and guidelines, especially for unexperienced clinicians. The following table resumes data from studies about different crestal approaches (Table 3).

Data from the present study seems to confirm the high predictability of this modified osteotome approach, when a residual crest at least 4 mm in high is present. In terms of implant design, in the present study, tapered implants were used. This design could be helpful when approaching the sinus by the residual alveolar bone crest, allowing for underpreparation of the implant site, and an osteotome effect during implant insertion. This study seems to confirm previous research in terms of MBL, involving similar implants (Nobel Replace tapered; [5,12]).

In the present research, anorganic bovine bone was used to graft the sinus. This material is widely reported in the international literature, including sinus lift procedures [5,12]. Some reflections could be made about eventual use of platelet-rich fibrin (PRF) or leukocyte- and platelet-rich fibrin (L-PRF) factors, instead of anorganic bovine bone, or in combination with it. Recently, a literature review by Mejia et al. concluded that there is not strong evidence about the advantages of the use of platelet concentrates in sinus lift [37].

To the best of our knowledge, this is the only study that describes the mixed approach with regular drill sequences and the use of only one osteotome. In conclusion, the results of the present study confirm that the main benefits of this approach are the easy technique, and the possible to use of it in association with any kind of implant, without any dedicated drill system, over a single osteotome.

## 5. Conclusions

Despite the limitations of the present retrospective study, it can be concluded that implants placed following a combined approach to the sinus consisting of sequential drills and osteotomes seem to be a viable treatment option for the rehabilitation of the posterior atrophic edentulous maxilla with a residual alveoli bone crest of 4 to 8 mm.

## Figures and Tables

**Table 1:** Exclusion criteria.

1	General contraindications for implant surgery
2	Lack of occluding dentition in the area intended for implant placement
3	Untreated periodontitis
4	Poor oral hygiene (Bleeding on probing (BOP) and/or plaque index (PI) > 25%)
5	Severe or moderate bruxism
6	Irradiation of the head and neck area in the previous five years
7	Uncontrolled diabetes
8	Heavy smoker (>10 cigarettes/day)
9	Substance abuse and/or psychiatric disorder
10	Pregnancy or lactation
11	Lack of 5-year post-loading data

**Table 2:** Main outcomes measurements.

	1 Year	5 Years
Survival rate	98.1%	98.1%
Complications	BOP at 2 implants	None
Marginal Bone loss	0.94 ± 0.18 mm	1.24 ± 0.28 mm

**Table 3:** Comparison with other similar studies.

Table 2	Implant Survival Rate	MBL	Follow-Up
Chen e Cha et al., 2005	99.3%	Not reported	8 years
Tallarico et al., 2017	100%	0.19 ± 1.05 mm	1 year
Xhanari et al., 2019	100%	0.99 ± 0.55 mm	1 year
Gatti et al., 2018	100%	0.33 ± 0.24 mm	2 years
Lumbau et al., 2020	98.1%	1.24 ± 0.28 mm	5 years

## Data Availability

The data presented in this study are available on request from the corresponding author.
